# Editorial: Emerging Diagnostic and Therapeutic Approaches for Gastric Cancer

**DOI:** 10.3389/fonc.2020.554960

**Published:** 2020-11-09

**Authors:** Kecheng Zhang, Sungsoo Park, Lin Chen

**Affiliations:** ^1^ Department of General Surgery, Chinese People’s Liberation Army (PLA) General Hospital, Beijing, China; ^2^ Department of Surgery, Korea University College of Medicine, Korea University Anam Hospital, Seoul, South Korea

**Keywords:** stomach cancer, minimally invasive surgery, multidisciplinary treatment, metastasis, prognosis

Gastric cancer is one of the most common malignancies worldwide. China and Korea contribute more than 50% of global new cases annually. Together with Park S from Korea, we focus on the topic of emerging diagnostic and therapeutic approaches for gastric cancer, as early detection and timely intervention are key measures to improve the poor prognosis of gastric cancer. In present collection, we receive a total of 58 submissions and a final of 20 articles composed by 173 authors are included. As of April 23th, this article collection has received 25,135 views.

Metastatic gastric cancer has a dismal prognosis and increasing attention has been paid to this group of patients. For metastatic gastric cancer, chemotherapy is the mainstay for therapeutic strategy, however, highly selected patients have improved survival after receiving multi-disciplinary treatment including gastrectomy. The controversies are that who may benefit from this multi-disciplinary gastrectomy, and when is the optimal time for surgical intervention. Five articles among this collection deal with these problems (Luo et al.; Zhang et al.; Zhao et al.; Sun et al.; Li and Zang). These studies identified risk factors for gastric cancer pulmonary metastasis and proposed surgical strategy for different category of gastric cancer with liver metastasis.

Tumor biomarker is of great clinical significance, because it facilitates decision-making for target therapy and helps to predict patients’ outcome. This useful biomarker includes circulating tumor cells, circulating tumor DNA, epidermal growth factor receptor family, and m6A RNA methylation, which have been discussed in the article collection (Yang et al.; Zhou et al.; Gao et al.; Arienti et al.; Su et al.).

Finally, we believe that this collection of review and original articles would contribute to early detection and management of gastric cancer. Translating these research findings into clinical practice may help improve survival of gastric cancer patients.

## Author Contributions

KZ composed the draft. LC and SP revised and approved the final version. All authors contributed to the article and approved the submitted version.

## Funding

This work was supported by grants from Program of Military Medicine for Youth (QNF19055) and National Natural Science Foundation of China (81972790, 81672319).

## Conflict of Interest

The authors declare that the research was conducted in the absence of any commercial or financial relationships that could be construed as a potential conflict of interest.

